# Natural History of Dilated Cardiomyopathy Due to *c.77T>C (p.Val26Ala)* in Emerin Protein

**DOI:** 10.3390/jcm13030660

**Published:** 2024-01-23

**Authors:** Néstor Báez-Ferrer, Felícitas Díaz-Flores-Estévez, Antonia Pérez-Cejas, Pablo Avanzas, Rebeca Lorca, Pedro Abreu-González, Alberto Domínguez-Rodríguez

**Affiliations:** 1 Cardiology Department, Hospital Universitario de Canarias, 38320 Tenerife, Spain; 2 Department of Genetics, Hospital Universitario de Canarias, 38320 Tenerife, Spain; fdiazflores@gmail.com (F.D.-F.-E.); aperezcejas@gmail.com (A.P.-C.); 3 Department of Laboratory, Hospital Universitario de Canarias, 38320 Tenerife, Spain; 4 Área del Corazón, Hospital Universitario Central Asturias, 33011 Oviedo, Spain; avanzas@secardiologia.es (P.A.); lorcarebeca@gmail.com (R.L.); 5 Instituto de Investigación Sanitaria del Principado de Asturias (ISPA), 33011 Oviedo, Spain; 6 Departamento de Medicina, Universidad de Oviedo, 33003 Oviedo, Spain; 7 Centro de Investigación en Red de Enfermedades Cardiovasculares (CIBERCV), 28029 Madrid, Spain; 8 Departamento de Biología Funcional, Área de Fisiología, Universidad de Oviedo, 33003 Oviedo, Spain; 9 Unidad de Cardiopatías Familiares, Área del Corazón y Departamento de Genética Molecular, Hospital Universitario Central Asturias, 33011 Oviedo, Spain; 10 Redes de Investigación Cooperativa Orientadas a Resultados en Salud (RICORs), 28029 Madrid, Spain; 11 Physiology Department, Faculty of Medicine, Universidad de La Laguna, 38200 Tenerife, Spain; pabreugonzalez@gmail.com; 12 Facultad de Ciencias de la Salud, Universidad Europea de Canarias, 38300 Tenerife, Spain

**Keywords:** dilated cardiomyopathy, emerin, titin, heart transplantation, malignant ventricular arrhythmia, heart failure

## Abstract

(1) **Introduction**: Dilated cardiomyopathy (DCM) mainly affects young individuals and is the main indication of heart transplantation. The variant *c.77T>C (p.Val26Ala)* of the gene coding for emerin *(EMD)* in chromosome *Xq28* has been catalogued as a pathogenic variant for the development of DCM, exhibiting an X-linked inheritance pattern. (2) **Methods**: A retrospective study was conducted covering the period 2015–2023 in patients with DCM of genetic origin. The primary endpoint was patient age at onset of the first composite major cardiac event, in the form of a first episode of heart failure, malignant ventricular arrhythmia, or end-stage heart failure, according to the presence of truncating variant in titin gene *(TTNtv)* versus the *p.Val26Ala* mutation in the *EMD* protein. (3) **Results**: A total of 31 and 22 patients were included in the *EMD* group and *TTNtv* group, respectively. The primary endpoint was significantly higher in the *EMD* group, with a hazard ratio of 4.16 (95% confidence interval: 1.83–9.46; *p* = 0.001). At 55 years of age, all the patients in the *EMD* group had already presented heart failure, nine presented malignant ventricular arrhythmia (29%), and 13 required heart transplantation (42%). (4) **Conclusions**: DCM secondary to the *c.77T>C (p.Val26Ala)* mutation in the *EMD* gene is associated to an increased risk of major cardiac events compared to patients with DCM due to *TTNtv*, with a large proportion of transplanted patients in the fifth decade of life.

## 1. Introduction

The estimated prevalence of heart failure (HF) is 2.1% among individuals between 45–65 years of age and is even somewhat higher among males [1]. Dilated cardiomyopathy (DCM) is defined as left ventricular or biventricular systolic dysfunction and dilatation that are not explained by abnormal loading conditions or coronary artery disease [2,3]. Advances in the diagnosis of DCM point to an estimated prevalence of between 1/250 and 1/400 individuals in the general population, often involving diagnostic confirmation based on the identification of a pathogenic genetic variant [4].

DCM mainly affects young individuals and is the principal indication of heart transplantation [5]. Pathogenic variants accounting for DCM have been found in over 60 genes, with truncating variant in titin gene *(TTNtv)* being the most commonly reported forms [5]. In 2012, they were estimated to be present in 25% of the familial presentations and in 18% of the sporadic cases of DCM [6]. Probably, the increase in identified mutations related to DCM has led to a decrease in the prevalence of *TTNtv* in DCM. Although the main mutations underlying familial DCM exhibit autosomal dominant inheritance, there have been descriptions of DCM with X-linked recessive inheritance [7]. The missense mutation *c.77T>C (p.Val26Ala)* in the gene of the emerin protein of the nuclear envelope *(EMD)* located in chromosome *Xq28* has been catalogued as a pathogenic variant for the development of DCM, exhibiting an X-linked inheritance pattern [8]. Advances in genetic technologies provide an opportunity to diagnose individuals based on their genetic findings, sometimes before clinical signs of the disease occur, allowing genetic counseling for family members [9].

The clinical course of patients with DCM due to the pathogenic variant *c.77T>C (p.Val26Ala)* in the *EMD* gene has not been previously described. The present study was carried out to define the clinical profile and the risk of suffering major cardiac adverse events in this particular population.

## 2. Methods

### 2.1. Study Population

This single-center, retrospective case-control study was conducted at Hospital Universitario de Canarias (Tenerife, Canary Islands, Spain) from 1 January 2015 to 31 June 2023. For the purpose of this investigation, we included patients from the Heart Failure Unit with DCM, which was defined as left ventricular or biventricular systolic dysfunction and dilatation that are not explained by abnormal loading conditions or coronary artery disease [2,3]. Patients presenting no described pathogenic variant for the development of DCM in the genetic study were excluded from the investigation. We analyzed all described pathogenic variants for DCM in the population and divided the sample into two groups. The case group consisted of patients with the pathogenic variant *c.77T>C (p.Val26Ala)* in the *EMD* gene, and the control group consisted of patients with *TTNtv* (nonsense, frameshift, splicing). In addition, we recruited the relatives carrying the pathogenic variant derived from the index case, but still with a negative phenotype. Likewise, the analysis excluded women carrying the pathogenic variant *c.77T>C (p.Val26Ala)* in the *EMD* gene, due to the X-linked recessive inheritance of this mutation.

At the time of identification of the corresponding pathogenic variant, we analyzed a number of variables such as clinical status, physical examination, and baseline variables (arterial hypertension, diabetes mellitus, ischemic coronary heart disease, and chronic kidney disease). We reviewed each of the complementary tests made during clinical follow-up, including electrocardiography, transthoracic echocardiography (TTE), cardiac magnetic resonance imaging (CMR), and cardiac catheterization. The blood samples for the genetic study were collected in the Genetics Unit of our center after obtaining due informed consent. The study protocol was approved by the Clinical Research Ethics Committee of Hospital Universitario de Canarias and was conducted in accordance with the principles of the Declaration of Helsinki. The authors of the study guaranteed anonymity of the collected data.

### 2.2. Genetic Analysis

The genetic analysis was carried out at Hospital Universitario de Canarias, which is certified for studies of this kind. Next-generation sequencing was applied, including genes for DCM and non-compaction cardiomyopathy (including the *EMD* gene and titin). The sensitivity and specificity of the NGS panel is over 99%.

### 2.3. Study Endpoints

The primary endpoint was patient age at onset of the first major cardiac event, which was a composite of first episode of HF, malignant ventricular arrhythmia (MVA), comprising sustained ventricular tachycardia, appropriate defibrillator therapy, or sudden cardiac death, and end-stage heart failure (ESHF), comprising bridging ambulatory inotropic treatment, the need for heart transplantation, or cardiovascular mortality.

The following secondary endpoints were established: (1) patient age at first HF episode; (2) patient age at first MVA; (3) patient age at classification as ESHF; (4) presence of atrioventricular conduction disturbances; (5) risk of composite MVA or ESHF according to left ventricular ejection fraction (LVEF) or the presence of late gadolinium enhancement (LGE) in CMR; and (6) clinical and cardiac structural differences in the initial clinical assessment of DCM patients carrying the pathogenic variant *c.77T>C (p.Val26Ala)* in the *EMD* gene with a positive phenotype versus patients with a negative phenotype.

### 2.4. Statistical Analysis

The baseline variables of both groups were reported as the mean ± standard deviation (SD) for quantitative variables and as frequencies (%) for qualitative variables. Comparisons of the variables were made using the Student *t*-test for quantitative data exhibiting a normal distribution, as confirmed by the Shapiro–Wilk test, while the nonparametric Wilcoxon rank sum test was used to compare variables exhibiting a non-normal distribution. Categorical variables were compared using the chi-square test, and statistical significance was considered for *p* < 0.05. The survival analysis of the groups was based on the plotting of Kaplan–Meier curves to describe the phenotypic expression of the different pre-specified cardiac events. Cox proportional hazards regression analysis or log rank tests were used to evaluate the association of clinical events according to the pathogenic variant involved. The data analysis was performed using Stata Statistical Software: Release 14 (StataCorp LP, College Station, TX, USA).

## 3. Results

### 3.1. Study Population

**Figure 1:**
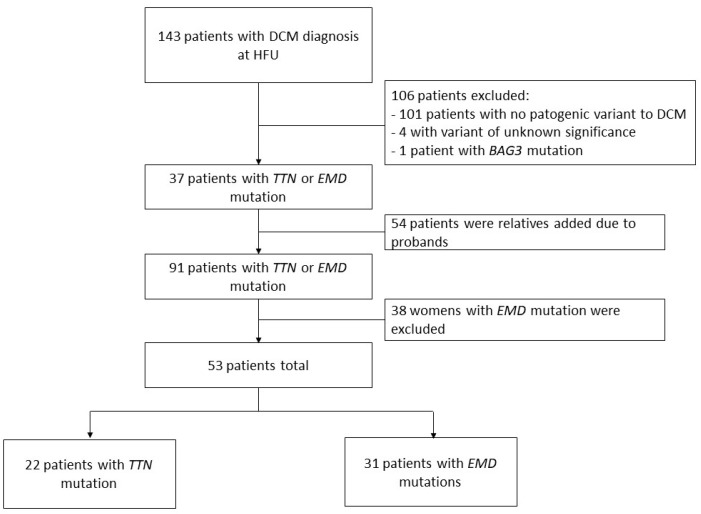
Study flowchart. Patient inclusion based on pathogenic variants. *BAG3*: BLC2-associated athanogene 3; DCM: Dilated cardiomyopathy; *EMD*: emerin; HFU: heart failure unit; *TTN*: titin.

The patient flowchart is shown in Figure 1. A total of 143 patients with DCM were identified in the Heart Failure Unit of our center. In 37 patients, we detected *TTNtv* as the cause of DCM or the pathogenic variant *c.77T>C (p.Val26Ala)* in the *EMD* gene. One patient with DCM due to a pathogenic variant of the *BAG3* gene and another four individuals with variants of undetermined significance were excluded, as were those yielding a negative genetic study (106 patients, Figure 1). In addition, we included 54 patients with familial segregation in some of the mentioned pathogenic variants. Thirty-eight women carrying the pathogenic variant *c.77T>C (p.Val26Ala)* in the *EMD* gene were excluded. A total of 53 patients were finally included in the investigation, 31 in the *EMD* group and 22 patients in the *TTNtv* group. We identified 20 families carrying the pathogenic variant *c.77T>C (p.Val26Ala)* in the EMD gene and 10 families with *TTNtv*.

### 3.2. Baseline Characteristics

The baseline characteristics of the 31 patients with DCM due to the pathogenic variant *c.77T>C (p.Val26Ala)* in the *EMD* gene are shown in Table 1. The median age of this total population was 42 years (interquartile range [IQR]: 34–47), and 11 were probands (35%). All patients with the DCM criteria were probands or relatives, but none of the relatives met the criteria of DCM. The median age of the patients with the DCM criteria at the time of first evaluation was 45 years (IQR: 41–49). There was only a limited presence of classical cardiovascular risk factors: arterial hypertension (14%), diabetes mellitus (10%), ischemic heart disease (10%), and chronic kidney disease (10%). None of these individuals presented skeletal myopathy. Electrocardiography evidenced complete left bundle branch block (LBBB) in 10 patients (48%), atrial fibrillation in nine (43%), a pathological Q-wave in five (24%), and prolongation of the PR interval in five subjects (10%). On the other hand, three patients (14%) were already carrying a permanent pacemaker at the time of diagnosis of this pathogenic variant in the *EMD* gene. Due to the previous presence of a pacemaker or implantable cardioverter-defibrillator (ICD) or implantable cardioverter-defibrillator and cardiac resynchronization therapy (ICD-CRT), five patients (24%) presented ventricular pacing with devices. Of note is the fact that none of the patients presented negative T-waves or complete right bundle branch block. The imaging techniques showed a median left ventricular end-diastolic diameter (LVEDD) of 60 mm (IQR: 59–61) and a mean diameter of 58 ± 5 mm, using TTE and CMR, respectively. The calculated LVEF was 35% ± 9% and 38% ± 10% using TTE and CMR, respectively. The left ventricular end-diastolic volume (LVEDV) quantified by CMR was 223 ± 44 mL. The mean right ventricular ejection fraction (RVEF) quantified by CMR was 52% ± 11%. There were no criteria of non-compaction cardiomyopathy in any of the patients. Globally, LGE was present in six patients (29%), with a subendocardial distribution in three subjects (14%), a transmural distribution in two (10%), and an intramyocardial distribution in one patient (5%), with no contrast uptake at the RV-LV junction in any case.

The patients without the diagnostic criteria of DCM at initial evaluation were significantly younger than those with the DCM criteria (median age: 25 years [IQR: 22–37] versus 45 years [IQR: 41–49]; *p* < 0.001). There were no statistically significant differences between the two groups in terms of the presence of classical cardiovascular risk factors such as arterial hypertension, diabetes mellitus, ischemic heart disease, or chronic kidney disease. Only one patient (10%) without the criteria of DCM presented LBBB, with an age at that time of 37 years. Another patient (10%) concomitantly presented atrial fibrillation and anteroseptal pathological Q-waves on the electrocardiogram at 44 years of age. Anterior hemiblock of the bundle of His was present in two patients (20%) aged 10 and 17 years. The presence of LBBB was more frequent in the group with the criteria of DCM (*p* = 0.03), and left anterior hemiblock was more frequent in the group that did not meet the criteria of DCM (*p* = 0.03). In statistical terms, atrial fibrillation tended to be more prevalent in the group with the criteria of DCM. Transthoracic echocardiography showed that the patients with the criteria of DCM had a greater LVEDD than those without the criteria of DCM (median LVEDD: 60 mm [IQR: 59–61] versus 45 mm [IQR: 45–55]; *p* < 0.01). Likewise, LVEF in the group of patients with the criteria of DCM was lower than in the group without the criteria of DCM (mean LVEF: 35% ± 9% versus 63% ± 7%; *p* = <0.0001). Cardiac magnetic resonance imaging was performed in only one patient (10%) without the criteria of DCM, with no presence of LGE, since most of the patients were very young individuals with no relevant structural cardiological alterations in the initial TTE study.

Compared with DCM due to *TTNtv* mutation, patients with DCM due to *c.77T>C (p.Val26Ala)* mutation in the *EMD* gene were younger and needed more ventricular pacing with devices. Pathogenic variants of DCM due to *TTNtv* mutation are described below: the following mutations were detected in a single patient: c.11554_11555delCA; c.55413delA (p.Gly18472Alafs); c.56541G>A (p.Tpr18847); c.60556C>T (p.Arg20186); two patients had c.24915_24916delGGinsTT (p.Gly8306) mutation; three patients had c.12028C>T (p.Gln4010) mutation; three patients had c.17086+1G>A mutation; four patients had c.27791_27792insT (p.Thr9265Asnfs) mutation, and six patients had c.37050G>A (p.Trp12350) mutation.

### 3.3. Phenotype Expression

**Figure 2:**
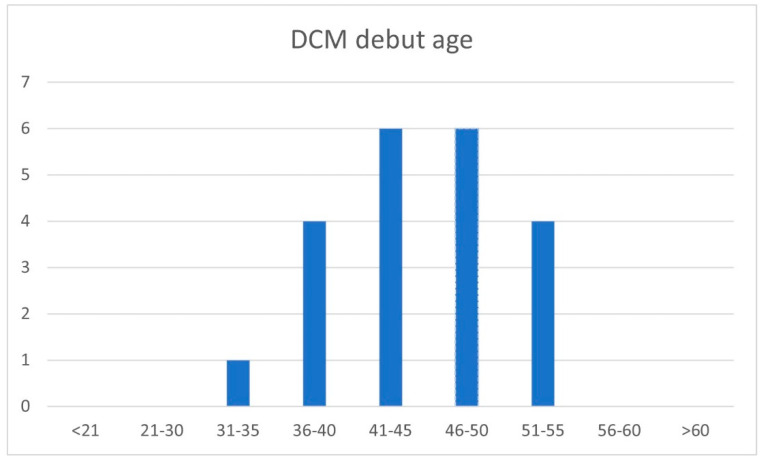
DCM debut age by five years. DCM: Dilated cardiomyopathy.

The age at presentation of the DCM phenotype in the group of individuals with the pathogenic variant *c.77T>C (p.Val26Ala)* in the *EMD* gene ranged from 34–53 years, with a median of 45 years (IQR: 41–49). None of the patients met the criteria of DCM at under 34 years of age. The percentage of patients presenting the criteria of DCM at < 21 years, 21–30 years, 31–35 years, 36–40 years, 41–45 years, 46–50 years, 51–55 years, 56–60 years, and >60 years was 0%, 5%, 19%, 29%, 29%, 19%, and 0%, respectively (Figure 2). All of the patients already presented DCM on entering the fifth decade of life. Nine patients presented MVA: three at between 35–40 years of age, five at between 41–50 years of age, and one at 52 years of age. In turn, 14 patients met the criteria of ESHF: 11 at between 41–50 years of age, and three at between 51–60 years of age.

### 3.4. Geographical Distribution

**Figure 3:**
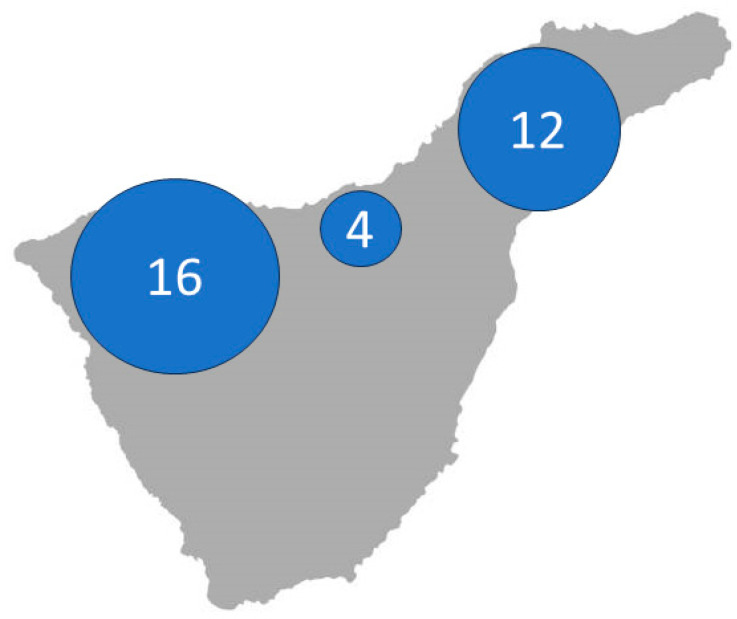
Number of patients with DCM due to the pathogenic variant *p.Val26Ala* in the *EMD* gene and its geographical distribution in North of Tenerife Island. DCM: Dilated cardiomyopathy; EMD: emerin.

The health care area of our center has been divided into three zones: the district of Daute to the west, that of La Orotava in the central zone, and the metropolitan area to the east, with populations of about 48,000, 121,000, and 217,000, respectively. The number of identified cases with DCM due to the pathogenic variant *c77T>C (p.Val26Ala)* in the *EMD* gene was distributed as follows: 16 cases in Daute, 5 cases in La Orotava and 12 cases in the metropolitan area. The disorder was clearly seen to predominate in the district of Daute, with a five-fold greater probability of finding DCM in this geographical setting (Figure 3).

### 3.5. Primary Outcome

**Figure 4:**
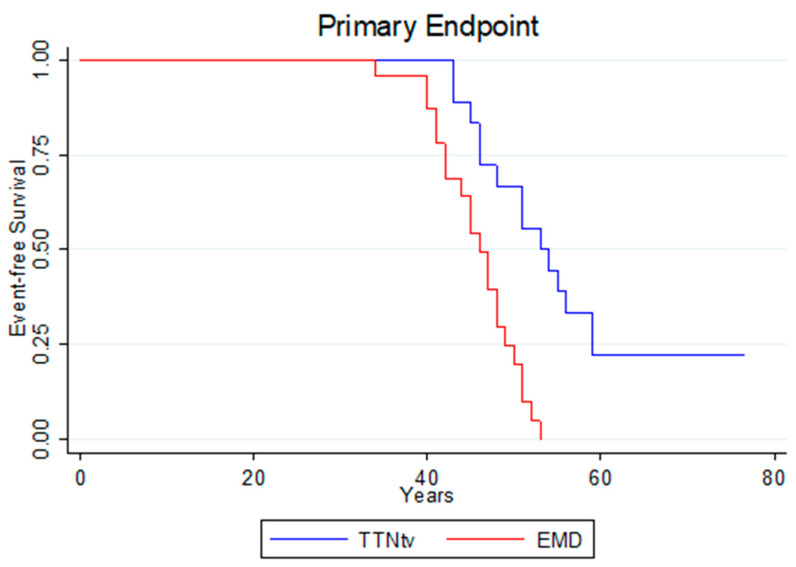
Primary endpoint, defined as a composite of first episode of heart failure, malignant ventricular arrhythmia, and end-stage heart failure. *EMD*: emerin; *TTNtv*: truncating variant in titin gene.

A total of 21 patients (68%) in the group of subjects with DCM due to the pathogenic variant *c.77T>C (p.Val26Ala)* in the *EMD* gene suffered the composite major cardiac event (onset of HF, MVA, or ESHF). In contrast, 14 patients (63%) with DCM due to *TTNtv* experienced the composite event. The presence of the composite major cardiac event was significantly greater in the *EMD* group, with a hazard ratio (HR) of 4.16 and 95% confidence interval (95% CI): 1.83–9.46; *p* = 0.001 (Figure 4).

### 3.6. Secondary Outcomes

#### 3.6.1. First Episode of Heart Failure

**Figure 5:**
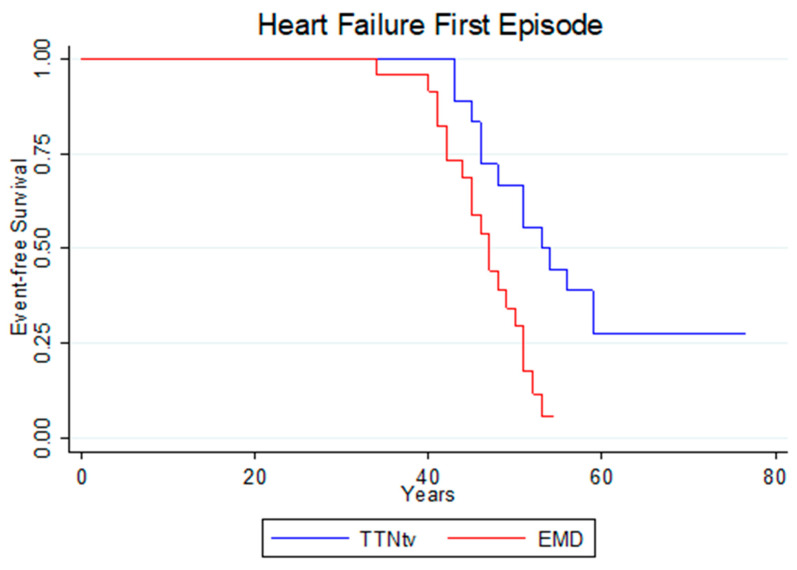
Age of Heart Failure First Episode. *EMD*: emerin; *TTNtv*: truncating variant in titin gene.

A total of 32 events were detected over follow-up in the two groups. Nineteen of them (61%) corresponded to patients with DCM due to mutation in the *EMD* gene, and 13 (59%) corresponded to patients with *TTNtv*. The risk of onset of HF was greater and manifested earlier in patients with DCM, due to the pathogenic variant *c.77T>C (p.Val26Ala)* in the *EMD* gene (HR: 3.05; 95% CI: 1.37–6.78; *p* = 0.006) (Figure 5). The presence of LVEF < 40% was not associated to a greater risk of prematurely experiencing the first HF event in patients with the pathogenic variant *c.77T>C (p.Val26Ala)* in the *EMD* gene (HR: 1.75; 95% CI: 0.66–4.63; *p* = 0.26).

#### 3.6.2. Malignant Ventricular Arrhythmia

**Figure 6:**
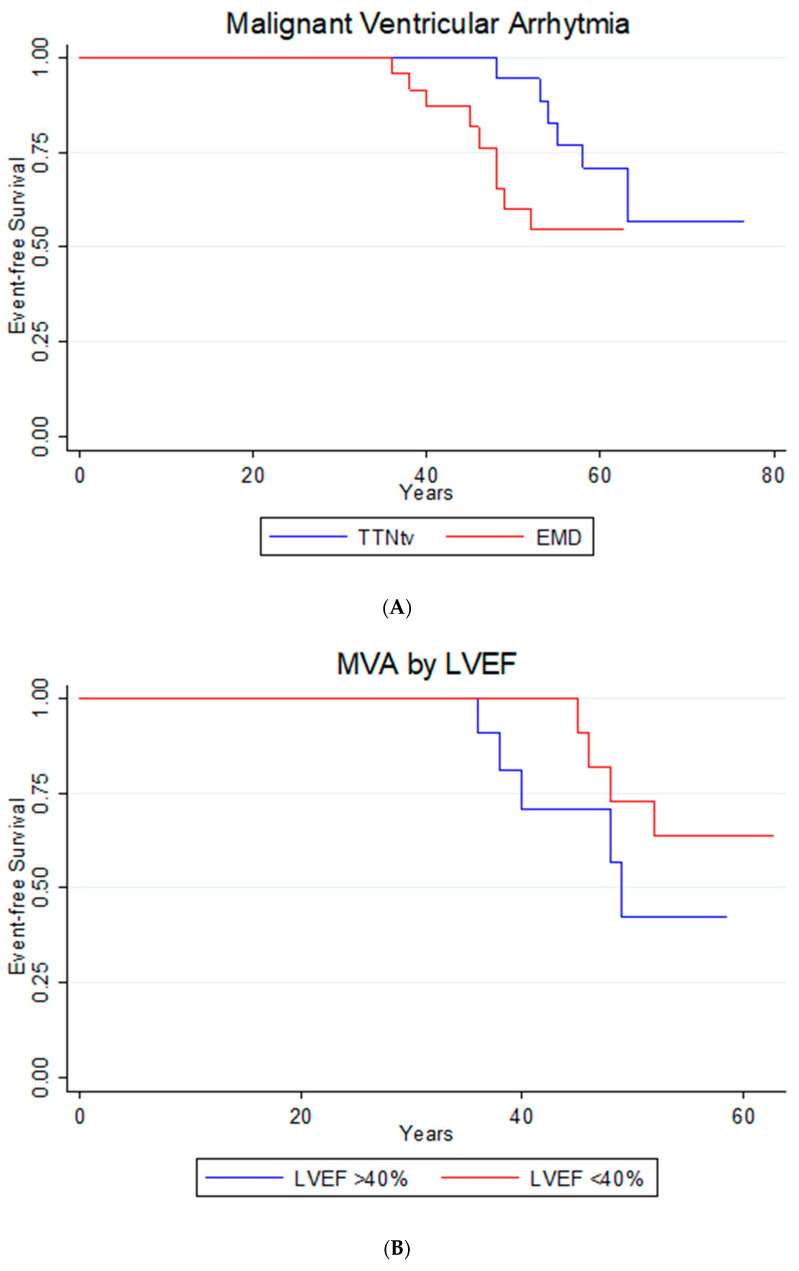

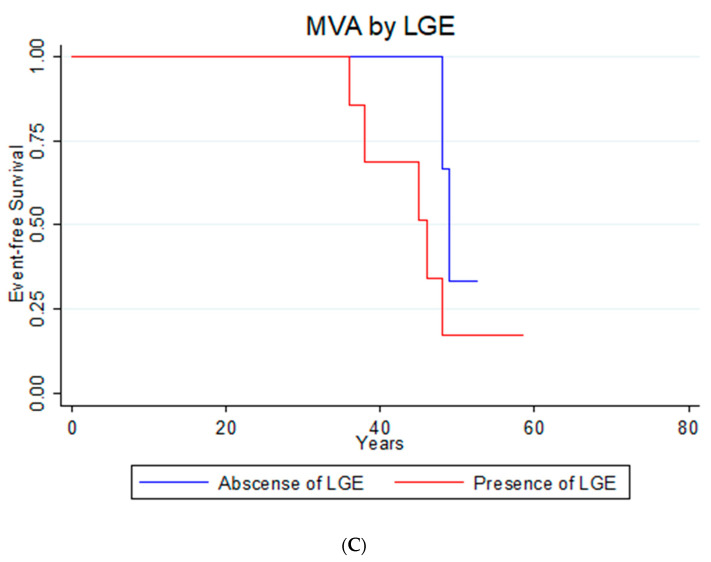
(**A**) Age of First Malignant Ventricular Arrhythmia defined as a composite of sustained ventricular tachycardia, appropriate defibrillator therapy, or sudden cardiac death. *EMD*: emerin; *TTNtv*: truncating variant in titin gene. (**B**) Malignant Ventricular Arrhythmia by left ventricular ejection fraction lower than 40%. MVA: Malignant Ventricular Arrhythmia; LVEF: Left Ventricular Ejection Fraction. (**C**) Malignant Ventricular Arrhythmia by presence or absence of late gadolinium enhancement in cardiac magnetic resonance. MVA: Malignant Ventricular Arrhythmia; LGE: Late Gadolinium Enhancement.

The retrospective follow-up identified 15 patients presenting MVA. Nine events (29%) corresponded to patients with DCM due to mutation in the *EMD* gene, and six (27%) corresponded to patients with *TTNtv* (Figure 6A). Despite the earlier manifestation of events in the group with the mutation in the *EMD* gene, there were no statistically significant differences (HR: 2.46; 95% CI: 0.81–7.47; *p* = 0.11). On analyzing the presence of MVA in the patients with DCM due to mutation in the *EMD* gene according to the presence of LVEF < 40% or LGE, no statistically significant differences were observed (HR: 0.44; 95% CI: 0.11–1.65; *p* = 0.22 and HR: 2.59; 95% CI: 0.48–13.93; *p* = 0.27, respectively). However, graphically there was a tendency towards a greater frequency and earliness of these episodes with LVEF > 40% and the presence of LGE at CMR (Figure 6B and Figure 6C, respectively).

#### 3.6.3. End-Stage Heart Failure

During follow-up, we identified 18 episodes of ESHF. Fourteen of the patients (41%) with DCM due to the pathogenic variant *c.77T>C (p.Val26Ala)* in the *EMD* gene presented this event. In contrast, it was only recorded in four patients (18%) with DCM due to *TTNtv*. The group with the mutation in the *EMD* gene had a greater risk of events over time than the *TTNtv* group (HR: 19.97; 95% CI: 2.59–153.91; *p* < 0.01). On analyzing the risk of suffering ESHF in the patients with DCM due to mutation in the *EMD* gene according to the presence or not of LVEF < 40%, we likewise recorded no statistically significant differences (HR: 1.28; 95% CI: 0.43–3.83; *p* = 0.66).

#### 3.6.4. Atrioventricular Conduction Disturbances

In the patients with DCM due to mutation in the *EMD* gene, four subjects (13%) presented atrioventricular conduction disturbances. Three patients required the implantation of a permanent pacemaker on identifying the pathogenic variant *c.77T>C (p.Val26Ala)* in the *EMD* gene, with the need for a subsequent upgrade to CRT-ICD over clinical follow-up. None of the patients with DCM due to *TTNtv* suffered atrioventricular conduction disturbances in our series.

## 4. Discussion 

To the best of our knowledge, this is the first study to describe the clinical course of patients with DCM due to the pathogenic variant *c.77T>C (p.Val26Ala)* in the *EMD* gene. In addition to DCM in the first decades of life, *Emery–Dreifuss* muscle dystrophy (EDMD) generates skeletal myopathy secondary to mutation in the *lamin A/C (LMNA)* gene with autosomal dominant inheritance or mutation in the *EMD* gene with X-linked recessive inheritance [10]. Our study shows that patients with the pathogenic variant *c.77T>C (p.Val26Ala)* in the *EMD* gene progress to a DCM phenotype during the fourth decade of life, with no cases being seen in younger subjects and with no diagnoses of skeletal myopathy—and moreover with high penetrance beyond the fifth decade of life. Likewise, this condition implies a high risk of major cardiac events, with a large proportion of patients ultimately requiring heart transplantation in the fourth or fifth decade of life (Graphical abstract).

The term non-ischemic DCM is very ambiguous, since it encompasses a range of etiologies (toxic, tachycardia-induced cardiomyopathy, or genetic) [4]. Genetic studies have played a key role in the etiological diagnosis of non-ischemic DCM, with the detection of a familial or hereditary origin in 20–50% of all cases. In addition, the disorder represents one of the main causes of HF and early sudden death among young patients [11]. Massive sequencing with large panels of genes has made it possible to identify new pathogenic genetic variants capable of causing cardiomyopathy. This is the case of the pathogenic variant *c.77T>C (p.Val26Ala)* in the *EMD* gene, which was identified by Cuenca et al. in 2016 in patients coming only from Tenerife (Canary Islands, Spain) [8].

The main gene related to DCM codes for the sarcomeric protein *titin*, representing 15–25% of all cases, and even 34% of all cases in selected familial DCM series [12,13]. Mutations in other proteins such as *myosin heavy chain 7* and *LMNA* follow in order of frequency, representing about 4–10% and 4–8% of all cases, respectively, depending on the series [12]. Most other mutations, apart from those mentioned above, are less common and are segregated into regional or familial groups, with DCM presenting X-linked recessive inheritance accounting for less than 1% of all cases [4,12]. During the last decade, it has been postulated that the DCM phenotype is not enough to establish a prognosis [14]. Precision medicine indicates that the DCM genotype can help us to predict the risk of cardiac events in patients, in conjunction with other classical clinical parameters [15]. In addition, the type of mutation can help predict arrhythmia risk, and particularly the risk of sudden death, with greater accuracy [13]. In general, the genes that code for proteins of the nuclear envelope are more associated with MVA and ESHF, and thus present a poorer prognosis [16]. In fact, the data suggest that mutations in the *LMNA* proteins of the nuclear envelope pose the greatest risk of major cardiac events and have the poorest prognosis [13,17]. Our results suggest that the pathogenic variant *c.77T>C (p.Val26Ala)* in the gene coding for the *EMD* protein of the nuclear envelope may prove similar to the mutations in the *LMNA* proteins as regards a poor patient prognosis. In fact, penetrance in carriers of mutations in the *LMNA* proteins may reach 61%, and this figure may prove even higher in our series of patients with mutations in the EMD gene, probably in relation to the X-linked recessive inheritance pattern.

### 4.1. Risk of Malignant Ventricular Arrhythmias

The arrhythmic risk due to mutations in sarcomere proteins and *titin* is less than in the case of mutations in non-sarcomere proteins (*desmoplakin*, *placophylin-2*, *LMNA* and *filamin C*). Specifically, the risk of MVA is much greater in the case of mutations in proteins of the nuclear envelope such as *LMNA* versus *TTNtv* [12,16]. In line with these observations, we found the pathogenic variant *c.77T>C (p.Val26Ala)* in protein *EMD* to be associated with greater arrhythmic events versus the population with *TTNtv*, though significant differences were not recorded, probably due to insufficient statistical power.

On the other hand, the phenotype may modify arrhythmic risk. Patients with DCM due to *TTNtv* and with reduced LVEF have a greater probability of suffering MVA [18]. In contrast, the risk of MVA and sudden cardiac death in patients with desmosomal gene and *LMNA* mutations is not modified according to LVEF [19]. However, the REDLAMINA registry recently showed that LVEF < 45% may indeed be associated to an increased arrhythmic risk [20]. In fact, the percentage of MVA in patients with *LMNA* mutations may vary between 18–50% depending on the series [17,21], being comparable to the 29% of MVA detected in our patients with the *EMD* gene mutation. Arrhythmic risk was not significantly modified in our series according to LVEF; however, there was a graphic tendency for LVEF > 40% and the presence of LGE at CMR to produce more and earlier MVA episodes. It is crucial in future to take into account the presence of LGE to stratify arrhythmic risk in each pathogenic variant, for it has already been shown that the combination of genetic positivity with the presence of LGE at CMR increases the prediction of MVA [22].

### 4.2. Risk of Atrioventricular Conduction Disturbances

In parallel, the proteins of the nuclear envelope, such as *LMNA*, are associated with the greatest risk of developing DCM and atrioventricular conduction disturbances. Any kind of cardiac conduction disturbance may be present in up to 73% of all patients with *LMNA* mutations [17], and up to 18% of the patients of the REDLAMINA registry presented third-degree atrioventricular block [20]. The existence of cardiac conduction disturbances has recently been described in patients with the pathogenic variant *c.77T>C (p.Val26Ala)* in the *EMD* gene [23]. The data of our series are comparable to those in patients with *LMNA* mutations, since three of the 21 individuals (14%) with the criteria of DCM at initial evaluation had evidence of third-degree atrioventricular block. Mutations in *LMNA* are also associated to LBBB [13]. In our patients with *EMD* gene mutations, 35% of the total and 48% of the individuals with the criteria of DCM at initial evaluation presented LBBB.

### 4.3. Heart Failure and End-Stage Heart Failure

The risk of HF and ESHF is greater in patients with DCM and *TTNtv* than in individuals with DCM and a negative genetic study. In fact, published data suggest that approximately 14% of all patients with *TTNtv* present the criteria of ESHF [18]. In our study, despite an 18% incidence of ESHF events, the risk of ESHF was much higher in the *EMD* mutation group. Mortality and the need for transplantation are higher in patients with *LMNA* mutations than in subjects without such mutations [24]. Mutations in *LMNA* pose the highest risk of mortality and need for heart transplantation versus other mutations in both sarcomere proteins and non-sarcomere proteins [13]. However, thanks to the Heart Failure and Familial Cardiopathy Units, many patients survive to heart transplantation, and in the same way as in the REDLAMINA registry [20], we only documented one death without being able to perform heart transplantation. The percentage of patients transplanted due to *LMNA* mutations may reach 27%; this figure is lower than in our series (45%) in patients with mutations in the *EMD* gene. The pathogenic variant *c.77T>C (p.Val26Ala)* mutation in the *EMD* gene is associated to an increased need for heart transplantation between the fourth and fifth decade of life, though this is not as early as in other mutations such as those of protein *RBM20*, where transplantation may prove necessary at around 28 years of age [17].

Further studies are needed to determine whether the usual medical treatment for HF with reduced LVEF can result in positive remodeling as may occur in DCM due to *TTNtv*, though mutations in desmosomal and nuclear envelope proteins are those associated with less LVEF recovery [12,16].

### 4.4. Molecular Hypothesis

*EMD* (34 kDa) is a 254-amino acid protein located on the cytoplasmic surface of the inner membrane of the nuclear envelope in cardiac muscle [10,25]. It interacts with the nuclear lamins, especially lamin A and nuclear actin, conforming a protein complex that regulates specific muscle genic expression [10]. Through its N-terminal LAP2-emerin-MAN1 (LEM) domain, it interacts with the DNA-binding protein barrier-to-autointegration factor (BAF) [26]. Recent studies in mice with a mutation in the nuclear envelope protein LEMD2 (p.L13R) generate dilated cardiomyopathy with a high burden of MVA and conduction disorders [27]. Other nuclear envelope proteins, such as TMEM43 (p.S358L), have been related to arrhythmogenic right ventricular cardiomyopathy, generating a high rate of MVA [28]. Interaction of the LEMD2 protein with the LEM domain and others suggests that mutations in nuclear envelope proteins generate a profile of patients with a higher risk of major cardiac events. In the cardiomyocytes of mice, through the growth factor pathways, the tyrosine kinase and protein G receptors are stimulated, activating the MAPK complex (mitogen factor-activated protein kinase) and stimulating transcription in fetal genes [25]. Negative regulation of MAPK may be altered in the presence of mutations in *EMD* and *LMNA* proteins [25].

In mice, it has been reported that β-catenin is a protein that regulates nuclear genic expression, modulating both positive and negative cardiac remodeling [25]. *EMD* binds to β-catenin, forming a protein complex which in the presence of EMD loss or malfunction may alter the β-catenin cycle and thus activate the transcription of genes implicated in cell hypertrophy and probably signaling pathways involved in overlap with DCM. β-Catenin is a component of the intercalated discs of the cardiomyocyte, and its alteration modifies the intercellular junctions; it has been suggested that this may be related to atrioventricular conduction disturbances [25,29]. These findings in mice suggest that therapeutic inhibition of the Wnt/β-catenin complex could afford benefit for patients with EDMD [30]. Furthermore, in vitro studies have shown that *EMD* is also closely related to β-dystroglycan, since the latter affords stability to *EMD* and positively regulates its function [31].

The earliness and severity of the EDMD phenotype is probably related to the absence of *EMD* protein. However, there are other mutations that affect the LEM domain, such as short deletion Lys37, where *EMD* protein is present but its spatial conformation is altered; as in this case, only atrial cardiac involvement may result [26]. Similarly, *p.Val26Ala* may generate a decrease in function of this protein, resulting in only cardiac involvement. However, further studies are needed in this specific field to clarify this relationship.

### 4.5. Study Limitations

This is a retrospective, single-center study. Due to the geographical island context involved in our sample, we have too few patients with *TTNtv* to allow more powerful statistical comparisons to be made. In many of these patients, genetic diagnostic confirmation was obtained after the diagnostic and therapeutic decisions of clinical practice, since the pathogenicity of the variant *c.77T>C (p.Val26Ala)* in the *EMD* gene was not known until 2016.

## 5. Conclusions

This is the first study to describe the cardiac events of DCM due to the pathogenic variant *c.77T>C (p.Val26Ala)* in the *EMD* gene. This DCM with X-linked inheritance implies an earlier and more premature onset of HF than in DCM due to *TTNtv*, as well as a greater risk of presenting the criteria of ESHF, with high penetrance in the fifth decade of life and a high heart transplantation rate between the fourth and fifth decade of life. Nevertheless, further studies on this pathogenic variant are needed in order to allow improved prevention and particularly the most personalized therapeutic guidance possible.

## Figures and Tables

**Table 1:** Baseline characteristics in *EMD* patients by DCM criteria at initial evaluation.

	Overall Patients with EMD Mutation (*n* = 31)	DCM at Initial Evaluation (*n* = 21) †	No DCM at Initial Evaluation (*n* = 10) ¥	*p*-Value ¥	Overall Patients with TTN Mutations (*n* = 22) ‡	*p*-Value ‡
Age *	42 (34–47)	45 (41–49)	25 (22–37)	<0.001	51 (46–56)	0.01
Proband patient	11 (35)	11 (52)	0 (0)	0.04		
Arterial hypertension	3 (10)	3 (14)	0 (0)	0.11	3 (14)	0.6
Diabetes mellitus	2 (6)	2 (10)	0 (0)	0.21	4 (18)	0.7
Ischemic coronary disease	2 (6)	2 (10)	0 (0)	0.21	0 (0)	0.07
Chronic Kidney Disease	2 (6)	2 (10)	0 (0)	0.21	0 (0)	0.07
Atrial fibrillation	10 (32)	9 (43)	1 (10)	0.055	4 (18)	0.06
Left bundle branch block	11 (35)	10 (48)	1 (10)	0.03	5 (23)	0.06
Right bundle branch block	0 (0)	0 (0)	0 (0)		1 (5)	0.3
Left anterior hemiblock	2 (6)	0 (0)	2 (20)	0.03	2 (9)	0.16
T-wave inversion	0 (0)	0 (0)	0 (0)		5 (23)	0.02
Q-wave	4 (13)	3 (24)	1 (10)	0.7	0 (0)	0.06
Long PR	2 (6)	2 (10)	0 (0)	0.21	0 (0)	0.13
Pacemaker	3 (10)	3 (14)	0 (0)	0.11	0 (0)	0.06
Paced	5 (16)	5 (24)	0 (0)	0.08	0 (0)	0.01
LV end-diastolic diameter by TTE *	59 (51–61)	60 (59–61)	45 (45–55)	<0.01	60 (51–64)	0.8
LVEF by TTE	45 ± 16	35 ± 9	63 ± 7	<0.0001	37 ± 15	0.7
LV end-diastolic volume by CMR		223 ± 44				
LV end-diastolic diameter by CMR		58 ± 5			59 ± 7	0.9
LVEF by CMR		38 ± 10			28 ± 11	0.1
RVEF by CMR		52 ± 11			48 ± 12	0.5
LGE	7 (23)	6 (29)	0 (0)	0.06	5 (23)	0.6
Subendocardial	3 (10)	3 (14)	0 (0)	0.11	0 (0)	0.06
Transmural	2 (6)	2 (10)	0 (0)	0.21	0 (0)	0.07
Intramyocardial	2 (6)	1 (5)	1 (10)	0.5	5 (23)	0.09
RV-LV junction	0 (0)	0 (0)	0 (0)		0 (0)	

Values are mean ± SD or median (interquartile range) for quantitative variables. Values are n (%) for qualitative variables. CMR: cardiac magnetic resonance; DCM: Dilated cardiomyopathy; LGE: Late Gadolinium Enhancement; LV: left ventricle; LVEF: left ventricle ejection fraction; RVEF: right ventricle ejection fraction; RV-LV: right ventricle–left ventricle; TTE: transthoracic echocardiography. *: variables with non-normal distribution; †: statistical reference group; ¥: comparison of “No DCM at initial evaluation” group with the reference group; ‡: comparison of “Overall patients with TTN mutations” group with the reference group.

## Data Availability

The data presented in this study are available on request from the corresponding author.
